# Evaluation of Optimal Control Approaches for Predicting Active Knee-Ankle-Foot-Orthosis Motion for Individuals With Spinal Cord Injury

**DOI:** 10.3389/fnbot.2021.748148

**Published:** 2022-01-24

**Authors:** Míriam Febrer-Nafría, Benjamin J. Fregly, Josep M. Font-Llagunes

**Affiliations:** ^1^Biomechanical Engineering Lab, Department of Mechanical Engineering and Research Centre for Biomedical Engineering, Universitat Politècnica de Catalunya, Barcelona, Spain; ^2^Health Technologies and Innovation, Institut de Recerca Sant Joan de Déu, Esplugues de Llobregat, Spain; ^3^Department of Mechanical Engineering, Rice University, Houston, TX, United States

**Keywords:** biomechanics, direct collocation, optimal control, spinal cord injury, knee-ankle-foot orthosis, exoskeleton, human motion prediction

## Abstract

Gait restoration of individuals with spinal cord injury can be partially achieved using active orthoses or exoskeletons. To improve the walking ability of each patient as much as possible, it is important to personalize the parameters that define the device actuation. This study investigates whether using an optimal control-based predictive simulation approach to personalize pre-defined knee trajectory parameters for an active knee-ankle-foot orthosis (KAFO) used by spinal cord injured (SCI) subjects could potentially be an alternative to the current trial-and-error approach. We aimed to find the knee angle trajectory that produced an improved orthosis-assisted gait pattern compared to the one with passive support (locked knee). We collected experimental data from a healthy subject assisted by crutches and KAFOs (with locked knee and with knee flexion assistance) and from an SCI subject assisted by crutches and KAFOs (with locked knee). First, we compared different cost functions and chose the one that produced results closest to experimental locked knee walking for the healthy subject (angular coordinates mean RMSE was 5.74°). For this subject, we predicted crutch-orthosis-assisted walking imposing a pre-defined knee angle trajectory for different maximum knee flexion parameter values, and results were evaluated against experimental data using that same pre-defined knee flexion trajectories in the real device. Finally, using the selected cost function, gait cycles for different knee flexion assistance were predicted for an SCI subject. We evaluated changes in four clinically relevant parameters: foot clearance, stride length, cadence, and hip flexion ROM. Simulations for different values of maximum knee flexion showed variations of these parameters that were consistent with experimental data for the healthy subject (e.g., foot clearance increased/decreased similarly in experimental and predicted motions) and were reasonable for the SCI subject (e.g., maximum parameter values were found for moderate knee flexion). Although more research is needed before this method can be applied to choose optimal active orthosis controller parameters for specific subjects, these findings suggest that optimal control prediction of crutch-orthosis-assisted walking using biomechanical models might be used in place of the trial-and-error method to select the best maximum knee flexion angle during gait for a specific SCI subject.

## Introduction

Walking impairment after spinal cord injury leads to a decreased quality of life, other serious health conditions (e.g., heart disease, high blood pressure), and substantial health care costs. Gait restoration can be partially achieved using active orthoses or exoskeletons, together with some type of external support for balance (e.g., crutches or a walker). In recent years, researchers have developed an active knee-ankle-foot orthosis (KAFO) for individuals with spinal cord injury that retain some hip mobility (Font-Llagunes et al., 2020). This assistive device locks the knee during the stance phase and imposes a pre-specified knee angle trajectory during the swing phase. Allowing knee flexion during swing, as opposed to a passive KAFO that locks the knee throughout the gait cycle, improves the gait pattern by increasing gait speed, stride length, and cadence while decreasing step width and lateral displacement of the center of mass (Font-Llagunes et al., 2020). In general, these modifications in gait patterns result in increased balance, reduced compensatory strategies, and decreased energy consumption (Michaud et al., 2019). To improve the walking ability of each patient as much as possible, it is important to personalize conveniently the parameters that define the device actuation, which may be different for each subject (Zhang et al., 2017; Cardona et al., 2020; Fricke et al., 2020) and may lead to undesired walking patterns if they are not correctly specified.

Exoskeleton controller parameters are usually manually adjusted based on subjective information, e.g., asking users which condition they prefer (MacLean and Ferris, 2019) or based on physiotherapists' visual assessment of basic gait parameters like foot clearance (Koopman et al., 2013). Personalization of the pre-specified orthosis knee motion parameter values for each spinal cord-injured (SCI) subject is typically done by following an experimental trial-and-error approach, which possesses significant limitations: (1) it is time-consuming and cumbersome for patients, (2) it is based on physiotherapist subjective intuition, (3) it necessitates training for physiotherapists when they start using this new device, and (4) it may lead to adverse events like falls if parameter values are not correctly specified. Because of these limitations, it is currently very difficult to select experimentally an optimal set of knee motion parameter values for a specific individual such that the assisted gait pattern is improved as much as possible. Therefore, a more objective way of evaluating different combinations of parameter values, which may also reduce required patient testing time, is needed.

Computational methods to identify assistive device design parameters or to tune their control parameters automatically have been developed in previous studies. In Fricke et al.'s study (Fricke et al., 2020), an algorithm to tune the assistance of a robotic gait trainer automatically was compared to manually-tuned assistance for 10 people with neurological disorders (six strokes and four spinal cord injuries). The authors concluded that automatic tuning of exoskeleton parameters is quicker than manual tuning and presents good performance, although clinical trials are needed to determine whether these apparent advantages result in better clinical outcomes. In Zhang et al.'s study (Zhang et al., 2017), a method for real-time identification of exoskeleton control parameters that minimize the metabolic energy cost of human walking was developed, and it was found that optimized assistance patterns varied widely across participants, demonstrating the importance of customization. Other studies used musculoskeletal models to estimate the user's kinetic parameters to control in real-time an exoskeleton (Cardona et al., 2020) or to simulate assisted human motion for identifying design parameters of assistive devices (García-Vallejo et al., 2016; Ong et al., 2016; Uchida et al., 2016). Moreover, optimal control has recently been used to identify the optimal spring characteristics of an ankle-foot orthosis that minimizes muscle effort (Sreenivasa et al., 2017), to predict subject-exoskeleton combined motion when lifting a box using a lower back exoskeleton (Millard et al., 2017), and to simulate a sit-to-stand transition using a lower limb exoskeleton (Serrancolí et al., 2019). Finding the correct optimal control problem formulation for the generation of new impaired or assisted walking motions is a current challenge (Mombaur, 2016; Falisse et al., 2019; De Groote and Falisse, 2021). In the study of Meyer et al. (2016), stroke patient walking was predicted at different speeds. In this work, in addition to minimizing joint jerk, the cost function included various tracking terms (upper body joint angles and lower body joint torques, muscle activations, or synergy activations), following the assumption that under different conditions the subject would try to find a solution close to what he did in the nominal case. In Sauder et al.'s study (Sauder et al., 2019), a personalized functional electrical stimulation treatment for fast-speed treadmill training was designed for an individual post-stroke. In that study, the cost function included minimization of joint jerk and minimization of inter-limb propulsive force asymmetry, which was the targeted gait improvement parameter. Finally, Febrer-Nafría et al. (2021) recently found that a multi-term cost function combining minimization of joint jerk, joint torque change, joint mechanical power, and angular momentum predicted four-point crutch walking well without tracking experimental data. All of these studies show that combining subject-specific models with optimal control methods is a promising approach to design patient-specific treatments, including the personalization of active orthoses for SCI subjects.

This study investigates whether the use of a computational approach to personalize pre-defined knee trajectory parameters for an active KAFO could potentially be a better choice than the current trial-and-error approach. This investigation does not compare directly the simulation approach with manual tuning but rather explores optimal control problem formulations that allow different pre-defined assistive knee angle trajectories to be simulated, thereby permitting identification of the best walking pattern for a specific individual with SCI. This goal has been pursued by using a new optimal control problem formulation for predicting crutch-orthosis-assisted walking of an SCI subject wearing the presented active orthoses, given as an initial guess the subject's gait without knee flexion-extension assistance (i.e., locked knee) and imposing a specific pre-defined knee angle trajectory. In this way, the experimental trial-and-error process of manually adjusting these knee motion parameters for each patient could potentially be avoided with improvement being estimated quantitatively. Such improvement has been defined in terms of change in clinically meaningful/relevant measurements, such as foot clearance, stride length, cadence, and hip flexion range of motion (ROM). In this work, the maximum knee flexion angle parameter, which is usually the first one tuned in the trial-and-error approach, has been investigated. Before applying the simulation pipeline to a specific SCI subject, the simulation pipeline was developed and evaluated using experimental data collected from a healthy subject. The main reason for using healthy subject data first was to enable a complete experimental evaluation of the predictive simulation approach. While the healthy subject could place each foot correctly on one force plate while walking using active orthoses and instrumented crutches, the SCI subject could not. First, an optimal control problem formulation for predicting crutch-orthosis-assisted walking was defined comparing different cost functions and evaluating them against experimental data (locked knee case for the healthy subject). Next, using the selected cost function, motions with knee flexion assistance for two different sets of knee angle trajectory parameter values were predicted, and changes in clinical measurements with respect to predicted locked knee motion were evaluated. Finally, using the selected cost function, locked knee motion and different motions with knee flexion assistance were predicted for an SCI subject. Given the subject's gait with passive supports, different knee trajectories (with four different maximum knee flexion angle parameters) were tested computationally. In this case, improvements in gait pattern were quantified in terms of changes in clinical measurements with respect to predicted locked knee motion. These results represent a step forward in the computational personalization of pre-defined knee angle trajectories for the control of an active KAFO for SCI subjects. We consider that having a simulation tool that allows testing of different pre-defined knee motions for a specific SCI subject model, with the aim of finding a more balanced and improved assisted gait pattern (with respect to the standard locked knee motion), will overcome the limitations of manual personalization of pre-specified knee motion parameter values and will result in an improved assisted motion for each SCI subject.

## Materials and Methods

### Orthosis Description and Current Personalization Methods

#### Orthosis Description and Function

**Figure 1:**
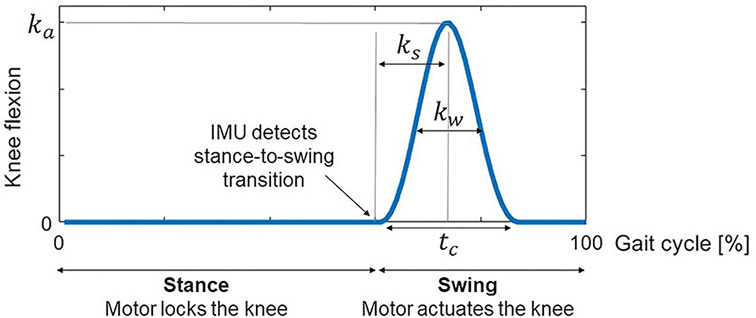
The active KAFO maintains the knee fully extended during the stance phase and provides knee flexion-extension assistance during the swing phase following a predefined knee flexion-extension trajectory. The parameters defining the knee flexion-extension trajectory are the following: *k*_*a*_ is the maximum knee flexion, *k*_*s*_ is the peak displacement parameter, *k*_*w*_ is the peak width parameter, and *t*_*c*_ is the cycle duration.

The active KAFO used in our study is intended for patients with SCI with some remaining motor function at the hip but who cannot control their knee and ankle muscles. These patients can walk using passive KAFOs (which avoid knee flexion and ankle dorsiflexion), which are custom-tailored to the subject, and crutches. However, the resulting gait is unnatural and exhausting due to the compensatory strategies associated with straight knee walking. The active KAFO provides knee flexion-extension assistance during the swing phase and maintains the knee fully extended during the stance phase, thanks to actuation provided by a brushless direct current motor combined with a harmonic drive transmission. In contrast to a passive KAFO, allowing knee flexion during swing improves the gait pattern by increasing balance, reducing compensatory strategies, and decreasing energy consumption (Michaud et al., 2019; Font-Llagunes et al., 2020). The active orthosis has a fixed ankle joint that keeps the foot perpendicular to the shank, and the length of the shank and the thigh links can be adjusted to fit the anthropometry of the user. Regarding the orthosis controller, inertial measurement unit (IMU) data are used to identify the time instant when the knee flexion-extension cycle must be triggered at swing phase initiation (stance-to-swing transition). Then, a proportional–integral–derivative (PID) control with feedforward velocity and acceleration is used to keep the knee in full extension during the stance phase (straight leg, knee locked) and perform a pre-defined knee flexion-extension trajectory after detection of the stance-to-swing transition (Font-Llagunes et al., 2020):


(1) $$\begin{array}{l} \theta \left(t\right)=\frac{k_{a}}{2}\left[1-\cos\left(\frac{2\pi }{t_{c}}t-k_{s}\sin\left(\frac{\pi }{t_{c}}t\right)-k_{w}\sin\left(\frac{2\pi }{t_{c}}t\right)\right)\right],\\ \;0\leq t\leq t_{c} \end{array}$$

where θ(*t*) is the pre-defined angle trajectory for each knee during the swing phase, *k*_*a*_ is the maximum knee flexion, *k*_*s*_ is the peak displacement parameter, *k*_*w*_ is the peak width parameter, and *t*_*c*_ is the flexion-extension cycle duration (Figure 1). The parameters defining the knee angle trajectory (*k*_*a*_, *k*_*s*_, *k*_*w*_ and *t*_*c*_) may be personalized to each subject so as to maximize their walking ability.

#### The Current Procedure for Personalizing Knee Angle Parameters

The current personalization method is based on manual tuning of the parameters that define the knee angle trajectory (Equation 1). Usually, the starting point is based on a default set of parameters that have worked for other patients. The patient walks with this set of values, and stride length and stance time for each leg are obtained from the device IMU measurements. In addition to this feedback, the physiotherapist measures relevant kinematic and spatiotemporal parameters that are usually used as outcome measures in clinical studies of lower-limb exoskeletons (Rodríguez-Fernández et al., 2021). One of the most important parameters is foot clearance, as the ankle of the orthosis is fixed at 90°. If there is not enough hip and knee flexion, the toes can catch the ground during the swing phase, which may produce a fall (Koopman et al., 2013; Begg et al., 2019; Di Natali et al., 2019; Fricke et al., 2020). Stride length and cadence are also important to indicate improvements in walking patterns (Koopman et al., 2013; Rasouli and Reed, 2020; Rodríguez-Fernández et al., 2021). Finally, hip flexion ROM is a good indicator of foot clearance and stride length (Cardona et al., 2020; Fricke et al., 2020; Rasouli and Reed, 2020; Rodríguez-Fernández et al., 2021). Moreover, bilateral symmetry in hip flexion is associated with bilateral symmetry in the gait pattern. Based on all of these clinical observations and measurements, some parameter values are modified in an iterative process based on the physiotherapist's experience. The first parameters that are modified are maximum knee flexion *k*_*a*_, which is the most critical one, and peak width *k*_*w*_. In some cases, especially if the patient presents some spasticity, the peak displacement parameter *k*_*s*_, which indicates flexion/extension duration ratio, is also modified.

### Development and Evaluation of the Prediction Framework Using Healthy Subject Data

In this work, experimental data were collected for two subjects, one healthy and one with SCI, both assisted by a pair of active KAFOs and a pair of forearm crutches. The simulation pipeline was developed and evaluated using the experimental data of the healthy subject and was later applied to predict SCI subject motion with different knee angle trajectories. A summary of the steps followed is provided below, and details regarding each step for the healthy subject are explained in this subsection, and for the SCI subject in the following subsection:

1) Collection of experimental data from the healthy subject
2) Computational model development for the healthy subject
3) Testing of different cost function formulations using healthy subject data with locked knee angle to identify the best formulation
4) Evaluation of the best cost function from step 3) using healthy subject data with different knee angle trajectories
5) Collection of experimental data from the SCI subject
6) Computational model development for the SCI subject
7) Evaluation of the best cost function from step 3) using SCI subject data with locked knee angle
8) Application of the best cost function from step 3) to predict SCI subject motion with different knee angle trajectories

#### Experimental Data Collection

**Figure 2:**
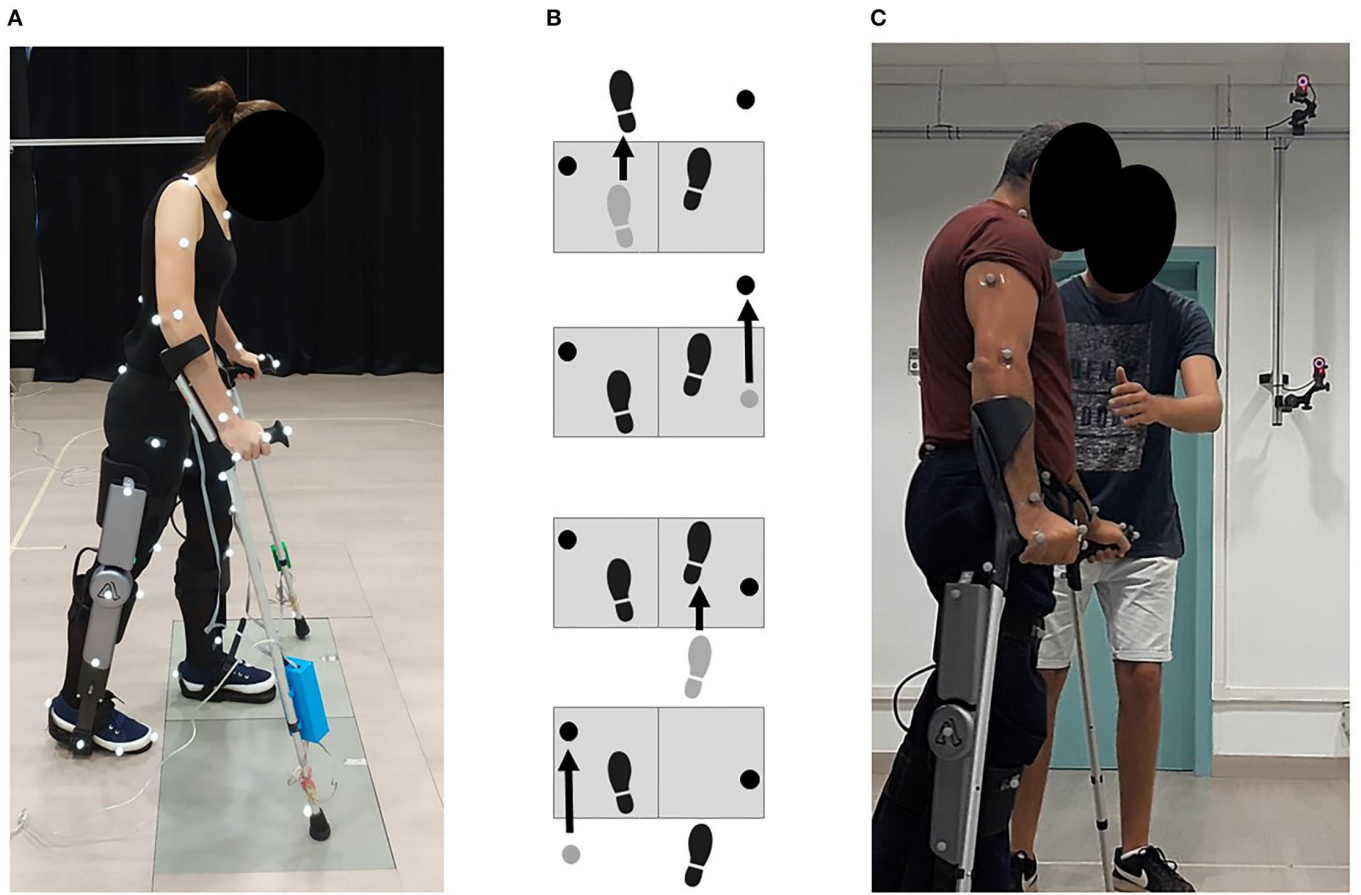
**(A)** Healthy subject experimental data collection. She walked in the transverse direction with respect to the force plates and used instrumented crutches. Three different trials were performed: one without knee flexion-extension assistance (locked knee), and two with different levels of maximum knee flexion during swing. **(B)** The followed crutch walking pattern in all trials was a four-point alternating pattern, being the swing phases sequence within the walking cycle: left crutch, right leg, right crutch, and left leg. **(C)** SCI subject experimental data collection. Only marker trajectories were collected for this subject. He walked with locked knees and non-instrumented crutches.

To find the most suitable problem formulation for predicting crutch-orthosis-assisted walking, we collected experimental gait data from a healthy subject walking with two active orthoses and crutches. The subject was a female (age 29 years, mass 54 kg, height 1.62 m) and the gait data were collected at the UPC Motion Analysis Laboratory in the Department of Mechanical Engineering of the Barcelona School of Industrial Engineering (ETSEIB) (Figure 2A). The reason for collecting experimental data from a healthy subject first was that it was easier for the subject to step correctly with one foot on each force plate while using wired instrumented crutches, thus providing a complete set of experimental data (synchronized marker trajectory, force plate, and crutch measurements). Moreover, orthosis kinematic performance is the same for a healthy subject as for a patient with SCI, since the knee controller follows a predefined flexion-extension angle and the IMUs detect the stance-to-swing transition event the same way in both cases. Three different trials were performed: one without knee flexion-extension assistance (locked knee) and two with different levels of maximum knee flexion during swing (35° and 45°). The crutch walking pattern used in all trials was a four-point alternating pattern with the following crutch placement sequence within the walking cycle: left crutch, right leg, right crutch, and left leg (Figure 2B). Collected data included marker trajectories, ground reactions (two force plates), and crutch forces (instrumented crutches). Surface marker motion was recorded at 100 Hz by tracking 53 passive reflective markers using 16 optical infrared cameras (OptiTrack V100:R2, NaturalPoint Inc., Corvallis, OR, USA). Ground reaction forces and moments were measured at the same sampling frequency by two force plates (AccuGait, AMTI, Watertown, MA, USA) located on the floor at the center of the capture workspace. Crutch-ground reaction forces were obtained from two custom-made instrumented crutches possessing 12 strain gauges each that sampled at 89 Hz (Sardini et al., 2015). Crutch data were interpolated to 100 Hz to match the sampling rate of the marker trajectory and force plate data.

#### Computational Model Development

A torque-driven model of the healthy subject with assistive devices was developed by incorporating a pair of forearm crutches and a pair of active orthoses into a currently available full-body model (Rajagopal et al., 2016) in OpenSim (Delp et al., 2007; Seth et al., 2018). The model possessed *n*_*q*_ = 31 degrees of freedom (DOF): six DOF between the pelvis and the ground (i.e., absolute translation and rotation), three for each hip, one for each knee, three for the lumbosacral joint, three for each shoulder, two for each elbow, and two for each wrist. Each DOF was associated with a model or joint coordinate *q*_*i*_ (*i* = 1, …, *n*_*q*_) in the order shown above, which formed the *n*_*q*_-dimensional vector of generalized coordinates ***q***. The model was scaled to the subject using the OpenSim Scale Tool, and a static trial was collected for this purpose. Each forearm crutch was incorporated into the model as a rigid body welded to the corresponding hand segment. The geometry and mass of each crutch were measured, and the crutch inertia tensor was obtained from simple rigid-body models. The active orthoses consisted of two segments (corresponding to thigh and shank-foot) with dimensions and inertial properties taken from CAD models of the real prototype. Each orthosis segment was attached to the corresponding lower limb segment using a weld joint (i.e., no relative motion was permitted between bodies). The ankle, subtalar and metatarsophalangeal joints were locked at 0° due to the presence of the orthosis mechanical constraints. No joints were defined between the orthosis links, i.e., the knee orthosis joint was considered to be perfectly aligned with the subject's knee joint. Despite being a simplification, this approach can be considered realistic as the orthoses were tightly attached to the subject with Velcro straps using front support at the shank and back support at the thigh and with a ratchet strap on each foot.

One representative gait cycle was selected for each condition (locked knee, 35 and 45° of knee flexion) and used in all subsequent model development and optimal control problem formulation tasks. The gait cycle was selected to use all available foot-ground reactions, which did not correspond to a complete crutch-gait cycle. The cycle started at left crutch off (LCO), and for the initial right leg stance phase, no force plate measurements were available. The subject walked in a transverse direction with respect to the force plates, which allowed clean placement of each foot on each force plate. Unfortunately, the subject was not able to place the crutches off the force plates at the same time (Figure 2B). This fact necessitated the crutch-ground reaction forces being subtracted from the force plate measurements to calculate the foot-ground reactions. The OpenSim Inverse Kinematics (IK) Tool was used to calculate joint coordinates for the full-body model (henceforth referred to as “experimental joint coordinates”). These joint coordinates and the measured foot- and crutch-ground reactions served as inputs to the OpenSim Inverse Dynamics (ID) Tool, which was used to obtain the “experimental joint torques.”

To model foot- and crutch-ground interactions, we used viscoelastic contact models whose parameter values were calibrated using an optimal control tracking problem. The foot-ground contact model consisted of 16 spring-damper units on each foot. The normal force in each element was generated using a linear spring with non-linear damping (Jackson et al., 2016), and the tangential force in each element was calculated using a simple continuous friction model (Jackson et al., 2016). The crutch-ground contact model consisted of a sphere at the tip of the crutch that could contact a plane representing the ground. The normal force was obtained using a Hertzian elastic point contact model with non-linear damping (Hunt and Crossley, 1975), and the tangential force model was the same as for the foot-ground contact model. The parameters of the foot-ground and crutch-ground contact models (i.e., spring stiffnesses, non-linear damping coefficients, dynamic friction coefficients) were calibrated by solving a direct collocation optimal control problem (Febrer-Nafría et al., 2021). The experimental locked knee trial motion and forces (joint angles, joint torques, and ground reactions) were tracked simultaneously while adjusting contact model parameter values that were assumed to be the same for both feet and both crutches.

#### Optimal Control Problem Formulations Comparison

Crutch-orthosis-assisted walking prediction problems were formulated as direct collocation optimal control problems (OCPs) using implicit skeletal dynamics (Van Den Bogert et al., 2011; Meyer et al., 2016; Falisse et al., 2019; Sauder et al., 2019) and were solved using the optimal control software GPOPS-II (Rao et al., 2014). Joint coordinates, velocities, accelerations, and torques were states in the problem, and joint jerk, joint torque change, and ground reactions were included as controls (Febrer-Nafría et al., 2021). Cycle duration, stride length, and relative duration of foot swing, crutch swing, and multiple support were considered free parameters in the optimization, and their values were bounded according to measured values (mean experimental value ± a specific tolerance). Initial guesses for all states and controls were the experimental values for the locked knee trial, as we considered that the prediction of assisted walking should be close to this initial motion.

The skeletal equations of motion obtained from OpenSim were included implicitly as algebraic path constraints (Van Den Bogert et al., 2011). An Inverse Dynamics (ID) analysis was performed at each iteration using the OpenSim C++ API (version 3.3), and the system kinematic state was used to calculate the generalized forces and torques (which included the six residual loads acting on the pelvis). Path constraints limited the residual loads to be within a specific tolerance (1 N, 1 Nm). The velocity of some specific points (midpoint for feet and tip for crutches) during the stance phase was bounded to avoid sliding. Periodicity was imposed for joint angles, joint torques, and normal contact forces. The symmetry between right and left foot-crutch mediolateral distance was imposed at the initial and final time. Mean speed for the pelvis anterior-posterior translation was limited within a specified tolerance.

Different cost function formulations were investigated based on published studies that predicted 3D full-body walking for clinical applications (Meyer et al., 2016; Sauder et al., 2019; Febrer-Nafría et al., 2021), considering only terms related to joint-level mechanics. Cost function terms were divided into three different groups: tracking terms (*J*_*track*_) that were closely reproduced, optimality terms (*J*_*opt*_) that were minimized, and regularization terms (*J*_*reg*_) that were also minimized (Equation 2):


(2) $$\begin{array}{l} J=\;\int_{t_{0}}^{t_{f}}\left(J_{track}\left(x,u\right)+\;J_{opt}\left(x,u\right)+0.01\;J_{reg}\left(u\right)\right)\;dt \end{array}$$

where *t*_0_ and *t*_*f*_ are the initial and final simulation times, respectively, ***x*** is the vector of states, and ***u*** is the vector of controls. Tracking terms included lumbar and hip flexion joint torque and upper limb joint angles; optimality terms included segment local angular momentum, joint mechanical power, and knee motor torque; regularization terms included joint jerk and joint torque change (Table 1). Different multi-term cost functions were implemented using two or more of these terms, and locked knee crutch-orthosis-assisted walking was predicted for each one of them. All cost function terms were scaled to be of a similar magnitude. To give more importance to the tracking and optimality criteria, we placed a weight of 0.01 on the regularization terms (minimization of joint jerk and minimization of torque change) for all combinations. Convergence and accuracy of simulation results predicted with each cost function were compared. Convergence was evaluated taking into account the number of iterations and computation time required to find an optimal solution, while accuracy was evaluated by computing the root mean square error (RMSE) between predicted and experimental joint angles and ground reactions. The cost function for which the best results were obtained was chosen to be used for all other crutch-orthosis-walking predictions generated in this study.

**Table 1:** Cost function terms considered in this work.

**Tracking terms** (*J*_*track*_)	Tracking of lumbar and hip flexion joint torque	$\sum_{i\;=\;\left\{1,5,9:11\right\}}{\left(\tau_{exp,i}-\tau_{i}\right)}^{2}$
	Tracking of upper limb joint angles	$\overset{n_{q}}{\underset{i\;=\;18}{\sum }}{\left(q_{exp,i}-q_{i}\right)}^{2}$
**Optimality terms** (*J*_*opt*_)	Minimization of segment local angular momentum	$\overset{n_{b}}{\underset{i\;=\;1}{\sum }}\;||L_{i}|{|}^{2}$
	Minimization of joint mechanical power	$\overset{n_{q}}{\underset{i\;=\;7}{\sum }}{\left({\overset{∙}{q}}_{i}\tau_{i-6}\right)}^{2}$
	Minimization of knee motor torque	$\sum_{i\;=\;\left\{4,8\right\}}\tau_{i}^{2}$
**Regularization terms** (*J*_*reg*_)	Minimization of sum of squared joint jerk	$\overset{n_{q}}{\underset{i\;=\;1}{\sum }}{{\overset{\ldots }{q}}_{i}}^{2}$
	Minimization of sum of squared joint torque change	$\overset{n_{q}-6}{\underset{i\;=\;1}{\sum }}{{\dot{\tau }}_{i}}^{2}$

*Tracking terms included (1) the sum of the squared error of lumbar and hip flexion joint torque, being τ_exp,i_ and τ_i_ the i^th^ component of the vector of experimental joint torques τ_exp_ and of predicted joint torques τ, respectively; and (2) the sum of the squared error of upper limb joint angles, being q_exp,i_ and q_i_ the i^th^ component of the vector of experimental joint coordinates q_exp_ and of predicted joint coordinates q, respectively. Optimality terms included (1) the sum of the squared norms of the segment angular momenta, being **n**_**b**_ the number of rigid bodies in the model and L_i_ the segment angular momentum at the center of mass of the i^th^ rigid body (or segment) of the model; (2) the sum of squared mechanical powers, computed for each lumbar joint coordinate, being ${\dot{q}}_{i}$ the i^th^ component of the vector of joint velocities $\dot{q}$, and **τ**_**i−6**_ the **(i−6)**^**th**^ component of the vector of joint torques **τ**; and (3) the squared value of the two knee torques, being τ_i_ the i^th^ component of the vector of joint torques τ. Regularization terms included (1) the sum of squared joint jerks, being ${\overset{\ldots }{q}}_{i}$ the i^th^ component of the vector of joint jerks $\overset{\ldots }{q}$; and (2) the sum of squared joint torque changes, being ${\dot{\tau }}_{i}$ the i^th^ component of the vector of joint torque change $\dot{\tau }$. To give more importance to the tracking and optimality criteria, a weight of 0.01 was placed on the regularization terms (minimization of joint jerk and minimization of torque change), for all the different combinations*.

#### Evaluation of the Selected Cost Function

To assess the ability of this prediction framework to perform virtual tests of pre-defined knee motion trajectories, we used the previously chosen cost function to predict assisted motions with knee flexion. In our simulations, we assumed that the IMU sensors detected correctly the stance-to-swing transition event and that the knee motor was capable of following the desired knee trajectory. Knee angle trajectory was defined according to Equation 1 using as maximum knee flexion *k*_*a*_ the exact value that was reached in the experimental trials. To quantify the performance of our simulation framework for testing virtually pre-defined knee motion trajectories, we computed RMSEs between predicted and experimental joint coordinates, joint torques, and ground reactions. Moreover, the following clinical measures that are usually used by physiotherapists in the trial-and-error process of manual tuning of knee angle trajectory parameters were computed: foot clearance, stride length, hip flexion ROM, and cadence. Foot clearance was obtained by computing the lowest vertical position of the toes' body origin in the OpenSim model during the swing phase for each foot. Stride length was computed as the mean value between feet and crutches stride length. Hip flexion ROM was computed as the right and left hip flexion ROM over the whole gait cycle. These clinical measures were evaluated by checking the improvements in assisted motions (with knee flexion) compared to locked knee motion.

### Application of the Prediction Framework Using SCI Subject Data

Once the crutch-orthosis-assisted walking prediction framework was developed and evaluated for the healthy subject, we applied it to test different pre-defined assistive knee angle trajectories for an SCI subject. This subject was able to walk with orthoses without knee flexion-extension assistance (locked knee) and non-instrumented crutches. We hypothesized that the same cost function would work for the SCI subject as for the healthy subject (Falisse et al., 2019), both for locked knee and flexed knee motions. We assumed that foot- and crutch-ground contact model parameters were the same as for the healthy subject, as the crutches and active orthoses used for both subjects were the same, and foot support of the orthoses contacted directly with the ground (i.e., the sole of the feet was the same for both subjects).

#### Experimental Data Collection

The subject selected for this study was a young adult male (40 years old, mass 72 kg, and height 1.72 m) that suffered paraplegia after a spinal hemangioma. He had an incomplete transverse spinal cord syndrome below the 10th thoracic neurological segment (T10), classified at level B in the ASIA Impairment Scale (AIS). Sensory but not motor function was preserved below the level of injury. During the experimental capture, it was difficult for the patient to walk with the instrumented crutches (as they included wires and electronic modules on each crutch) and to place one foot cleanly on each force plate. For these reasons, the experimental capture was done with non-instrumented crutches, thus collecting only marker trajectories. One trial with locked knees was recorded to be used as an initial guess for the different prediction problems (Figure 2C).

#### Computational Model Development

A torque-driven model of the SCI subject with crutches and active orthoses was developed following a similar approach as for the healthy subject. To take into account the SCI subject impairment, we limited hip joint torque production according to the functional state of the subject. Following an approach similar to Sreenivasa et al. (2017), we assumed that the SCI subject used 80% of his hip motor capacity during the experimental capture with passive orthoses and crutches. Another difference with respect to the healthy subject model was that for the SCI subject, the total torque acting at the knee corresponded to the assistive motor torque since the subject's knee muscles were not functional. This torque was limited to ±34 Nm, which is the peak torque that the electric motor can provide. To obtain the reference values for hip joint torques, we solved an optimal tracking problem that tracked joint coordinates obtained from IK while minimizing joint jerk. Ground reactions were obtained from foot-ground and crutch-ground contact models using the parameter values obtained for the healthy subject. Hip joint torques were then limited assuming that the reference values obtained from this tracking problem were 80% of the maximum values.

#### Evaluation of the Selected Cost Function

To evaluate the convenience of using the same cost function for predicting locked knee crutch-orthosis-assisted walking for an SCI subject, we computed the RMSE between predicted and experimental joint angles (obtained after IK analysis).

#### Knee Motion Strategy Testing

Knowing the subject's gait with locked knees, we computationally tested different knee angle trajectories, having all parameters fixed and modifying the knee maximum flexion parameter *k*_*a*_. This parameter is usually the one that is varied first in the trial-and-error adaptation process. Four different motions were predicted with the following levels of knee flexion: 20, 30, 40, and 50°. Joint torques and ground reactions obtained from the locked knee tracking problem were used as the initial guess for the predictive simulations. To evaluate the performance of our simulation framework for testing virtually pre-defined knee motion trajectories for an SCI subject, we computed foot clearance, stride length, hip flexion ROM, and cadence, and we compared these clinical measures among all predicted motions. The maximum knee flexion value that produced the most improved walking motion was selected as the best candidate for this SCI subject.

## Results

Different cost functions for predicting locked knee crutch-orthosis-assisted walking were explored, combining one or more tracking, optimality, and regularization terms. Overall, we found that minimizing join jerk helped the problem converge more quickly and satisfied mesh error tolerance at the first mesh iteration. We also found that minimizing lumbar joint mechanical power, segment angular momentum (especially torso), and motor torque helped the problem converge with fewer iterations and lower joint angle errors. Moreover, we found that having joint torque as a state and joint torque change as a control worked better than having joint torque as a control. Based on these results, we chose the following cost function for predicting crutch-orthosis-assisted walking: minimization of lumbar mechanical power, all segment angular momentum, motor torque, joint jerk, and joint torque change. Using this cost function, an optimal solution for the healthy subject with locked knee was found in 27 min, and mean RMSE was 5.34° for joint angles, 55.40 N for normal forces (feet and crutches), and 14.35 N for tangential forces (feet and crutches) (Table 2). Overall, the lowest mean RMSEs for all joint angles were found for the healthy subject with locked knee motion (5.74°), followed by the SCI subject with locked knee motion (8.19°), and finally the healthy subject with 45° knee flexion (9.45°) and 35° knee flexion (10.26°) (Table 2). For the healthy subject, higher errors were found for upper limb joint angles, whereas for the SCI subject, errors were higher for the lower limbs.

**Table 2:** Convergence (number of iterations and computation time) and accuracy (mean RMSE for angular coordinates and ground reactions) for all predictive simulations.

	**Subject**		**Healthy**			**SCI**				
	**Trial**		**H0**	**H35**	**H45**	**S0**	**S20**	**S30**	**S40**	**S50**
Convergence	N iterations		322	759	582	153	125	197	990	414
	Computation time		27 min	2 h 26 min	1 h 51 min	13 min	25 min	40 min	3 h 12 min	1 h 23 min
Mean RMSE	Angular coordinates [°]	Pelvis + torso	4.88	6.95	6.24	10.67	-	-	-	-
		Upper limbs	6.46	12.65	10.76	5.74	-	-	-	-
		Lower limbs	5.13	8.57	9.57	10.61	-	-	-	-
		All	5.74	10.26	9.45	8.19	-	-	-	-
	Ground reactions [N, Nm]	Normal	55.40	90.71	53.84	-	-	-	-	-
		Tangential	14.35	15.19	13.03	-	-	-	-	-
		Moment	6.90	11.07	9.58	-	-	-	-	-

*The name for each trial indicates the subject (“H” for healthy and “S” for SCI subject), followed by the maximum knee flexion angle in degrees. For the SCI subject, only errors in angular coordinates for the locked knee case (S0) were computed, according to the available experimental data*.

**Figure 3:**
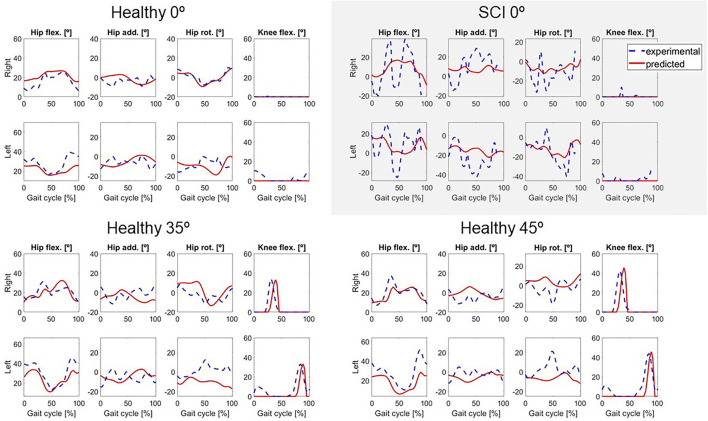
Predicted lower limbs joint coordinates (in red) for locked knee motion (0°) for both subjects, and for two active knee flexion motions for the healthy subject, compared to the experimental values (in dashed blue).

**Figure 4:**
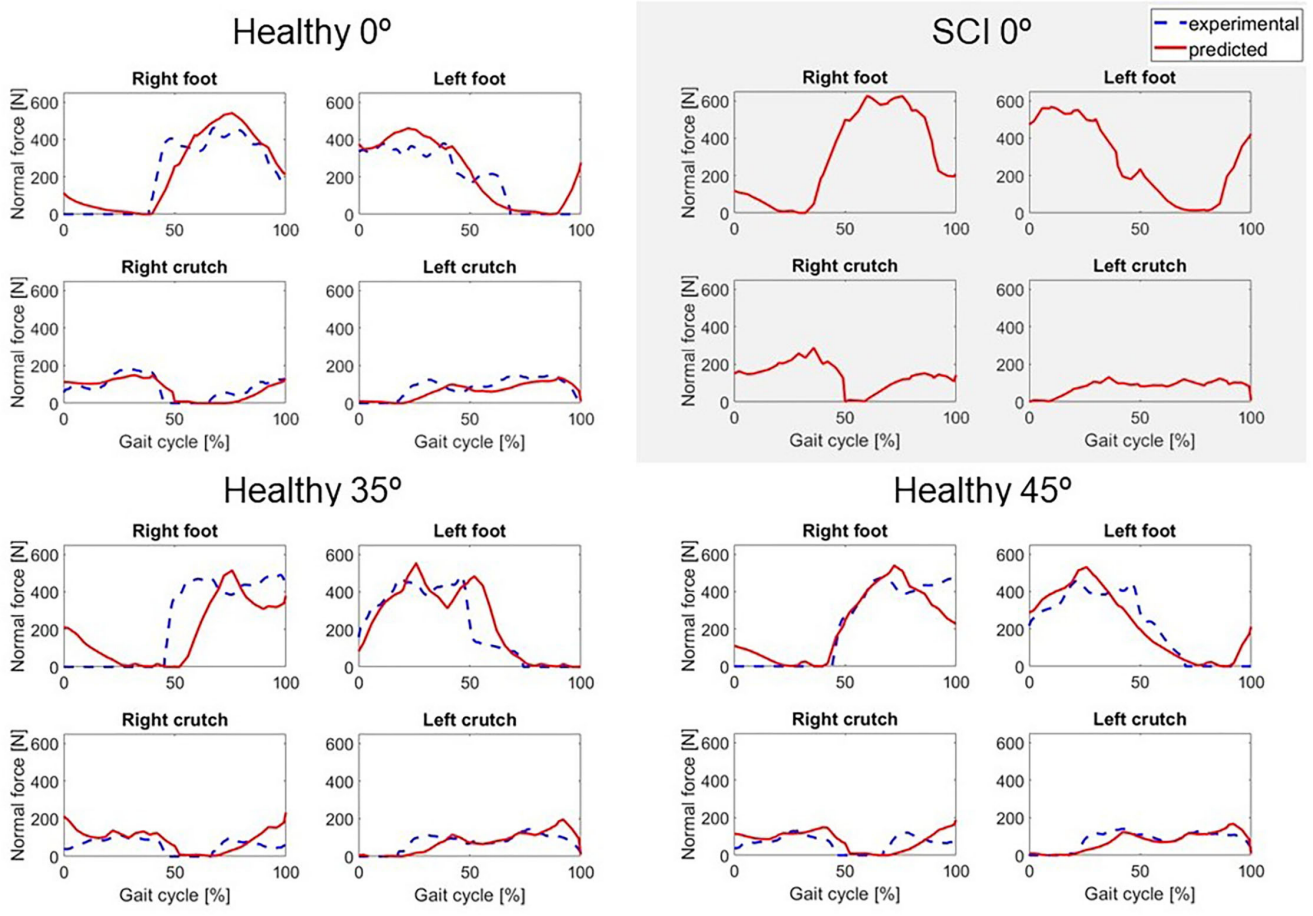
Predicted normal forces (in red) for locked knee motion (0°) for both subjects, and two active knee flexion motions for the healthy subject, compared to the experimental values (in dashed blue). For the SCI subject, no force plate measurements were available.

Two different crutch-orthosis-assisted gait cycles were predicted for the healthy subject (with maximum knee flexion values of 35 and 45°, respectively), imposing the knee angle trajectory from the collected experimental trials. We assumed that the same cost function would work for predicting both locked knee and flexed knee-assisted walking. The computation time required to converge was higher than for the locked knee case, up to 2 h 30 min (Table 2). Mean RMSE for joint angles was 10.26° for 35° of predicted knee flexion motion and 9.45° for 45° of predicted knee flexion motion. In both cases, the lowest mean RMSE was for pelvis and torso joint angles (<7°) and the highest was for upper limbs angles (10.7–12.6°). Hip flexion was predicted better than were hip adduction and hip rotation, with predicted angle trajectories showing peaks at the same cycle times as in the experimental data (Figure 3). Regarding ground reaction forces, the mean RMSE for normal forces was higher for 35° knee flexion motion (90.71 N) compared to locked knee motion but was similar for 45° knee flexion motion (53.84 N) (Table 2). Errors in tangential forces were comparable for locked knee and both flexed knee motions (13–15 N). In all cases, predicted foot and crutch weight-bearing was consistent with the experimental ground reaction forces (Figure 4).

**Figure 5:**
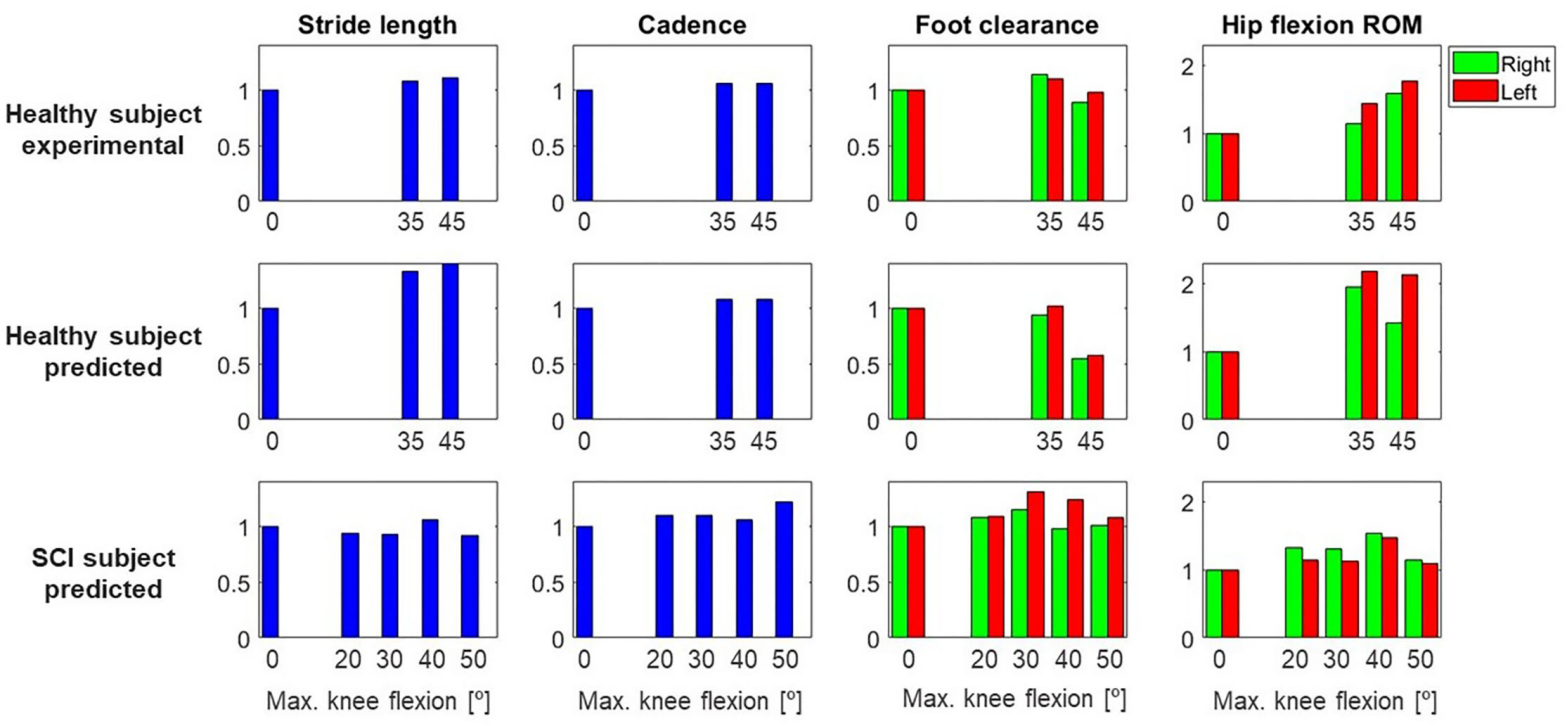
Gait parameters (stride length, cadence, foot clearance, hip flexion ROM) for both subjects (healthy, SCI) and each different maximum knee flexion angle, normalized to the locked knee (0°) case. This normalization was done for each subject and case (experimental and/or predicted), and for right and left sides independently. For the healthy subject, experimental and predicted values are shown. For the SCI subject, only predicted values are shown, as no data were collected for the different maximum knee flexion conditions. Note that the maximum value in the y-axis is higher for hip flexion ROM than for the other parameters.

Changes in foot clearance, stride length, and cadence were in general well-predicted for the healthy subject. When comparing flexed knee motions with respect to locked knee motions, foot clearance increased for 35° and decreased for 45° in both experimental and predicted motions (Figure 5). Stride length and cadence increased for both flexed knee motions, and stride length was higher for 45° compared to 35° in both experimental and predicted motions. Compared to locked knee motion, hip flexion ROM also increased for flexed motions in experimental and predicted motions. However, there was a difference between the trend in predicted motions compared to experimental motions: in the experimental motions, hip flexion ROM increased for 35 and 45° with respect to the locked knee case, but in the predicted motions, it increased for 35° and decreased for 45°.

In general, the locked knee predicted gait pattern for the SCI subject had a lower ROM for each joint coordinate (Figure 3) and less mediolateral movement (in the frontal plane) compared to experimental measurements. Stride length and cadence were comparable in experimental and predicted motions (0.51 m for both motions and 30.23 steps/min and 27.26 steps/min, respectively). Foot clearance was higher, and symmetry improved in the predicted motion (4.82 and 4.72 cm, for right and left foot, respectively, compared to 3.60 and 1.50 cm), whereas hip flexion ROM was lower, and symmetry decreased in the predicted motion (26 and 17°, for right and left hip, respectively, compared to 58 and 54°). Regarding ground reactions, predicted normal forces were higher for the right side (peak value of 0.75 body weight (BW) for right foot and 0.34 BW for right crutch) compared to the left side (peak value for 0.68 BW for left foot and 0.16 BW for left crutch) (Figure 4).

Four additional crutch-orthosis-assisted gait cycles were predicted for the SCI subject with maximum knee flexion angle increasing from 20 to 50° in increments of 10°. Optimal solutions were found in a mean time of 1 h 20 min, with a maximum computation time of 3 h 12 min (Table 2). In general, compared with locked knee predicted motion, all four clinical measures improved for predicted motions with knee flexion (Figure 5). Maximum values of foot clearance (right: 5.56 cm, left: 6.16 cm) were found for 30° of maximum knee flexion, maximum values for stride length (0.54 m), and hip flexion ROM (right: 40.09°, left: 26.31°) were found for 40° of maximum knee flexion, and maximum value for cadence was found for 50° knee flexion (33.37 steps/min).

## Discussion

In this work, we developed an optimal control prediction approach to test different pre-defined knee angle trajectories of an active orthosis to assist the gait of SCI subjects. We compared different cost functions and chose the one that produced results closest to experimental locked knee crutch-orthosis-assisted walking for a healthy subject. Having as an initial guess the experimental motion for the locked knee case, we predicted crutch-orthosis-assisted walking imposing knee flexion using different maximum knee flexion parameters to define the knee angle trajectory along the gait cycle. For the healthy subject, two different maximum knee flexion angles were imposed for which experimental walking data were available. For the SCI subject, no experimental walking data for flexed knee motion were available, and four different maximum knee flexion angles were imposed in the simulations. We evaluated changes in four simulated clinical measures that are usually considered by physiotherapists to decide the best set of parameters for a specific patient (foot clearance, stride length, cadence, and hip flexion ROM). These changes were consistent with those observed in the experimental motions for the healthy subject and were reasonable for the SCI subject. These findings suggest that it may be beneficial to use optimal control predictions of crutch-orthosis-assisted walking in place of the current trial-and-error method to select the best maximum knee flexion angle for a specific SCI subject.

Changes in the clinical measures were generally predicted well for the healthy subject. For the healthy subject collected motions, we found that having knee flexion-extension assistance produced better results for stride length, hip flexion ROM, and cadence, as all of these measures increased for both maximum knee flexion angles (35 and 45°) compared to the locked knee case. These changes are related to improved assisted motion and are linked, as having increased knee flexion is associated with having increased hip flexion (Escalante et al., 1999), and generally increased hip flexion is correlated with a longer stride length (Schulz et al., 2008). For the predicted motions, having knee flexion assistance also produced increased values for hip flexion ROM, stride length, and cadence. In the case of stride length and cadence, the trend observed in experimental measurements was also observed in the predicted motions (higher values for 45° compared to 35°). However, in the case of hip flexion ROM, higher values were obtained for 45° in experimental motions and 35° in predicted motions. Thus, this trend in hip flexion was not well-captured by the optimal control problem, though in both experimental and predicted motions with knee flexion assistance, hip flexion ROM was higher than in the locked knee case. Moreover, the asymmetry between right and left hip flexion ROM observed in the experimental motions (left hip flexion ROM was higher than right hip flexion ROM) was also observed in the predicted motions. Foot clearance with respect to the locked knee case increased in the experimental motions for 35° but decreased for 45°. This trend was also observed in the predicted motions, where left foot clearance increased for 35° (and right foot clearance remained almost equal) and both feet clearance decreased for 45°.

Considering the four clinically relevant measures, our results suggest that for this particular SCI subject, 30 or 40° of maximum knee flexion may produce the best-assisted motion. Compared to locked knee prediction, foot clearance generally increased for knee flexion assistance. This outcome is desirable when using lower limb exoskeletons, since straight knee gait and drop foot gait reduce foot clearance (Koopman et al., 2013; Yeung et al., 2018). For both legs, the best case was for 30°, which produced foot clearances of 5.56 and 6.16 cm (right and left, respectively) compared to 4.82 and 4.72 cm (same order) in the locked knee case. Stride length was in general slightly lower for flexed knee motions compared to locked knee motion but slightly increased for 40°. Hip flexion ROM clearly increased for all flexed knee motions. The highest values were obtained for 40° and the lowest for 50°. There was also asymmetry in hip flexion ROM, with the right hip flexion ROM being higher, as was also observed in the experimental motion. For 40°, the highest values of hip flexion ROM and stride length were obtained. Cadence increased slightly for all flexed knee motions, with the highest value of 33.37 steps/min occurring for 50° compared to 27.26 steps/min in the locked knee predicted motion. These results are reasonable if we relate them to knee kinematics during normal walking. In normal gait, the knee flexion-extension cycle starts at a terminal stance and ends at a terminal swing (Perry, 1992). During knee flexion, the ankle dorsiflexes, which increases foot clearance, and during knee extension, the ankle eccentrically plantar flexes as the cycle enters terminal swing. In our simulations, it should be noted that only the maximum knee flexion was modified for the different predictions, meaning that the flexion-extension cycle had the same allotted time to reach maximum knee flexion across all the conditions. As a result, the knee flexion-extension motion was faster for the 50° condition compared to the 30° condition and may have impacted hip flexion, leading to a shorter stride length. However, the increased speed of the knee flexion-extension cycle may have created a momentum effect and therefore led to a higher cadence.

Evaluating the assisted motion and choosing the best set of parameters for a specific subject, manually or computationally, is not always straightforward. In this work, compared to locked knee motion, we found for both subjects that clinical measures improved for assisted motions with knee flexion until a certain peak knee flexion was reached (around 35° for the healthy subject and between 30 and 40° for the SCI subject), with results worsening with higher knee flexion values. This trend was observed both experimentally (for the healthy subject) and computationally (for both subjects) and coincided with what the authors have observed in different training sessions with SCI subjects wearing the lower limb active orthosis. However, it is not clear how these clinical measures should be interpreted, e.g., if some of them improve while others do not for the same set of parameters. Usually, exoskeleton parameters are manually adjusted based on subjective evaluation, e.g., asking users which condition they prefer (MacLean and Ferris, 2019) or based on physiotherapists' visual assessment on basic gait parameters like foot clearance (Koopman et al., 2013). For our active orthosis, some objective values are added to the physiotherapist's subjective evaluation, as the device provides real-time feedback of stride length and weight-bearing time on each leg. However, the evaluation is still done subjectively by the physiotherapist, who decides how to tune the orthosis parameters manually based on both subjective assessment and objective measurements. Simulation (or automatic tuning) presents some advantages compared to trial-and-error tuning: it is quicker (Fricke et al., 2020), and many parameter sets can be virtually tested without the risk of trying a combination that will not work and could be harmful to the patient. Despite these advantages, there is no clinical evidence to date that the automatic tuning of assisted motions results in better clinical outcomes (Fricke et al., 2020). It is difficult to develop a system that objectively takes into account all of the factors that a physiotherapist evaluates whilst assisting a patient to walk. It could be that for a specific parameter set, some important clinical measures improve while others do not, and one clinical parameter could be more critical for one patient than for others. Before this method can be applied to choose optimal knee control parameters for a specific subject, more research is needed to understand better and define objectively the targeted assisted gait pattern for the patient according to functional status.

Although trends in clinical measures were well-predicted for the healthy subject, in some cases absolute predicted values differed from experimental values. Cadence was well-predicted: 37 steps/min and 40 steps/min in locked and flexed knee motions, respectively, in both experimental and predicted results. For foot clearance, a lower value was generally found for predicted motions compared to experimental motions, with the highest difference being 2.10 cm for the left leg with a 45° maximum knee flexion angle. Stride length and hip flexion ROM were also lower in predicted motions compared to experimental motions. Stride length was 14 cm lower for predicted locked knee motion compared to experimental conditions (0.51 vs. 0.37 m) and 4–5 cm lower for predicted flexed knee motions. Hip flexion ROM was up to 12° lower in predicted vs. experimental conditions for locked knee motion and up to 18° lower for flexed knee motion. These reductions in stride length and hip flexion ROM were mainly caused by lower joint angle ROMs in predicted motions compared to experimental motions (Figure 3), which also resulted in lower foot clearance.

Differences in joint angles and ground reaction forces between predicted and experimental motions were lowest for the healthy subject with locked knee condition (that is, for the case for which the cost function was selected). This finding may indicate that different cost functions should be used for locked/flexed knee and healthy/SCI subjects, or, if healthy and pathological human gaits emerge from similar control strategies (Falisse et al., 2019), that the appropriate cost function has not yet been identified, and other terms should be added that may play an important role only for the SCI subject. Further research is needed to determine the best cost function for predicting assisted walking of SCI subjects. This effort will require the collection of a complete set of experimental data (including marker trajectories and foot- and crutch-ground reactions). Moreover, the best cost function for simulating crutch walking may be different than the best one for simulating normal walking. As far as the authors know, there is only one study that predicts crutch-assisted walking using a 3D full-body model (Febrer-Nafría et al., 2021). In the SCI subject lower limbs, only the hip is actuated by muscles, and the hip ROM was not well-predicted. This result may be caused by the fact that hip motion was controlled by net torque actuators instead of individual muscle-tendon units. Meyer et al. (2016) found that predicting walking under new conditions was more accurate if muscles rather than net torque actuators were used to generate the motion, with muscles controlled by synergies instead of individual muscle activations producing the most accurate walking predictions. Therefore, we hypothesize that including muscles in the model and controlling them by synergies could improve the prediction results. However, for that approach, it would be challenging to calibrate muscle-tendon model parameter values for patients with SCI. Moreover, when we tested whether adding more tracking terms might improve the predicted motions (Meyer et al., 2016), we did not find a clear improvement compared to cost functions without tracking terms. Given our results, we believe that adding targeted tracking terms could produce more subject-specific assisted motions following a pattern closer to the one chosen initially by the patient with a locked knee. Even though the cost function requires further investigation, these results are promising as we have been able to predict changes in crutch-orthosis-assisted walking motions that are in good (for the healthy subject) or reasonable (for the SCI subject) agreement with experimental trends. Our hypothesis is that by finding a cost function that predicts the locked knee condition better, we will be able to predict walking motions with knee flexion assistance more reliably, and changes from locked to flexed knee conditions will be maintained.

This work possesses several limitations. First of all, no training or learning process was performed by the healthy subject, and experimental data were collected before the subject was used to walking with the active orthoses. Therefore, some clinical measures could be different after such a training process. Moreover, only two different levels of maximum knee flexion were tested for the healthy subject, and the maximum value was the only knee angle trajectory parameter varied. For the clinical application, the assisted gait of a single SCI subject was simulated, and only one of the parameters that define the knee angle trajectory was explored. Although maximum knee flexion is the most critical parameter, it would also be interesting to predict how varying the other three parameters would affect the predicted motion. In addition, we assumed that the stance-to-swing transition event was perfectly detected during the simulation. Gait event detection is done using IMUs in the real device, and some threshold parameters need to be adjusted as well. In future experiments, we will start with some training sessions, and we will collect data for different values of all parameters that define the knee angle trajectory and gait event detection. In this way, we will be able to assess if some parameters are more subject-dependent and others more general. Another limitation was that we did not directly compare manual tuning using a trial-and-error process with the computational or simulation tuning. This comparison process would be complex, as different aspects should be taken into account: (1) time and effort of the physiotherapist to find these values, (2) time and effort of the patient, (3) if different physiotherapists find different values, and (4) if both methods produce similar results in terms of the better-assisted motion. A benefit of using a computational model would be to obtain a personalized default set of parameters that could then be easily tuned in the clinic. This approach would reduce fitting time and would be safer for the patient, as there would be less risk of adverse events than when using non-personalized parameters. An adverse event like a fall would have a big impact on the patient's health and confidence in the technology. We hypothesize that in general, the set of parameters provided by the model will work well for the patient, though there will always be particular cases where the manual tuning of this initial set of parameters will be needed. These cases include patients who fatigue over the session or have changing levels of spasticity or pain. These aspects are currently not considered in the model, and therefore, it might be necessary to tune the initial set of parameters to accommodate these issues. In future work, how to include fatigue, spasticity, or pain in the model should be investigated. Another potentially complicating factor is that the cost function might vary with time. Regarding the active KAFO modeling, in this work, the knee motor modeling was simplified. We assumed that the knee flexion angle trajectory was followed correctly, and that maximum knee flexion was reached. However, we observed experimentally that maximum flexion was lower than the targeted value. In future work, we will investigate how to include actuator dynamics (Nguyen et al., 2020). Finally, we considered the same knee flexion angle trajectory for both right and left legs, but asymmetry was observed in the trials in both healthy and SCI subjects. In the future, we will investigate if the assisted knee flexion trajectory should be different for both legs to achieve a more symmetric gait pattern.

In conclusion, this study explored the feasibility of using a computational approach to personalize the pre-defined knee trajectory parameters for an active KAFO for SCI subjects. We developed an optimal control prediction approach to test different pre-defined knee angle trajectories of an active orthosis to assist the gait of SCI subjects. We checked if our optimal control approach was capable of correctly predicting assisted motions for different values of maximum knee flexion angle, evaluating results against experimental data collected from a healthy subject assisted by the active orthoses. While trends in clinical measures were well-predicted, absolute predicted values differed from experimental values in some cases. We applied the framework to predict assisted gaits of an SCI subject with four different maximum knee flexion values. To the best of the authors' knowledge, no study in the literature has addressed how to formulate optimal control problems to predict novel crutch-orthosis-assisted walking motions using 3D full-body models. Although more research is needed before this method can be used to choose optimal knee control parameters for a specific subject, our findings suggest that optimal control prediction of crutch-orthosis-assisted walking using biomechanical models might possess benefits over the current trial-and-error method used to select the best maximum knee flexion angle for a specific SCI subject. Having a simulation tool that allows different pre-defined knee motions to be tested on a specific SCI subject model, with the aim of finding a more balanced and improved assisted gait pattern (with respect to the standard locked knee motion), could overcome limitations of the current manual personalization process and could yield an improved assisted motion for each SCI subject.

## Data Availability Statement

The datasets presented in this article are not readily available because participants have not given written informed consent to make them publicly available. Requests to access the datasets should be directed to miriam.febrer@upc.edu.

## Ethics Statement

The studies involving human participants were reviewed and approved by Research Committee of Universitat Politècnica de Catalunya. The patients/participants provided their written informed consent to participate in this study. Written informed consent was obtained from the individual(s) for the publication of any potentially identifiable images or data included in this article.

## Author Contributions

MF-N ran the experimental data collection sessions, processed all experimental data, performed all modeling work, formulated and ran all optimal control problems, and wrote a first draft of the manuscript. JF-L and BF planned and supervised the entire project, assisted with the development of optimal control problem formulations, evaluated optimal control results, and helped with revising the manuscript. All authors contributed to the article and approved the submitted version.

## Funding

This work was conducted with support from the Grants DPI2015-65959-C3-2-R and RTI2018-097290-B-C33 funded by MCIN/AEI/10.13039/501100011033 and by “ERDF A way of making Europe”, and the Leonardo Grant for Researchers and Cultural Creators 2018 funded by the BBVA Foundation.

## Conflict of Interest

The authors declare that the research was conducted in the absence of any commercial or financial relationships that could be construed as a potential conflict of interest.

## Publisher's Note

All claims expressed in this article are solely those of the authors and do not necessarily represent those of their affiliated organizations, or those of the publisher, the editors and the reviewers. Any product that may be evaluated in this article, or claim that may be made by its manufacturer, is not guaranteed or endorsed by the publisher.
